# Tracking and prevalence of cardiovascular disease risk factors across socio-economic classes: A longitudinal substudy of the European Youth Heart Study

**DOI:** 10.1186/1471-2458-6-20

**Published:** 2006-01-27

**Authors:** Peter L Kristensen, Niels Wedderkopp, Niels C Møller, Lars B Andersen, Charlotte N Bai, Karsten Froberg

**Affiliations:** 1 Institute of Sports Science and Clinical Biomechanics, University of Southern Denmark, Campusvej 55, 5230 Odense M, Denmark; 2 The Back Research Center, Lindevej 5, 5750 Ringe, Denmark; 3 Department of Sports Medicine, Norwegian School of Sport Sciences, Postboks 4014 Ullevål Stadion, 0806 Oslo, Norway

## Abstract

**Background:**

The highest prevalence of several cardiovascular disease risk factors including obesity, smoking and low physical activity level is observed in adults of low socioeconomic status. This study investigates whether tracking of body mass index and physical fitness from childhood to adolescence differs between groups of socioeconomic status. Furthermore the study investigates whether social class differences in the prevalence of overweight and low physical fitness exist or develop within the age range from childhood to adolescence.

**Methods:**

In all, 384 school children were followed for a period of six years (from third to ninth grade). Physical fitness was determined by a progressive maximal cycle ergometer test and the classification of overweight was based on body mass index cut-points proposed by the International Obesity Task Force. Socioeconomic status was defined according to *The International Standard Classification of Occupation *scheme.

**Results:**

Moderate and moderately high tracking was observed for physical fitness and body mass index, respectively. No significant difference in tracking was observed between groups of socioeconomic status. A significant social gradient was observed in both the prevalence of overweight and low physical fitness in the 14–16-year-old adolescents, whereas at the age of 8–10 years, only the prevalence of low physical fitness showed a significant inverse relation to socioeconomic status. The odds of both developing and maintaining risk during the measurement period were estimated as bigger in the group of low socioeconomic status than in the group of high socioeconomic status, although differences were significant only with respect to the odds of developing overweight.

**Conclusion:**

The results indicate that the fundamental possibilities of predicting overweight and low physical fitness at an early point in time are the same for different groups of socio-economic status. Furthermore, the observed development of social inequalities in the absolute prevalence of overweight and low physical fitness underline the need for broad preventive efforts targeting children of low socioeconomic status in early childhood.

## Background

Cardiovascular diseases (CVD) are a major health problem in the industrialized world. More than 40% of all deaths in Denmark (24.156 of 60000 in 1998) are caused by CVD. The highest prevalence of several CVD risk factors including obesity, smoking and low physical activity level is observed in adults of low socio-economic status (SES). [1-3]. In fact, CVD show a relatively clear socio-economic gradient in adults [4-7]. Naturally, this gives rise to concern not only as regards the health of adults of low socio-economic status but also as regards their children. Parents have been shown to be effective teachers of health habits, and children's lifestyle, health beliefs and behaviour are significantly influenced by their parents' behaviour [8]. A number of studies have been designed to investigate the association between socio-economic status and coronary heart disease risk factors in children and adolescents. However, the results have so far been inconsistent – thus the picture is less clear than concerning adults [9,10]. It has been suggested that this inconsistency could be, at least partly, due to differences in the ages of the populations being studied but further investigations are necessary [9].

In order to work out effective preventive strategies against CVD it is important to determine at which stage in life the relatively clear socio-economic gradient in obesity, as well as other CVD risk factors, observed in adults start developing. Furthermore, due to the expected changes in lifestyle/risk status between groups of SES at some point in childhood or adolescence, it is appropriate to examine the stability of risk factors within different groups of SES. It is often advocated that an important aspect of the preventive effort against CVD is to identify subjects at risk of developing CVD at an early point in time, so preventive strategies or clinical treatment can be applied at early stages. The rationale behind this preventive strategy is that measurements of risk factors for CVD early in life predict values later in life. Several studies have described tracking of CVD risk factors in children [11-14]. However, to our knowledge, no study has analysed and compared tracking of CVD risk factors among children from families of different socio-economic status and thereby posed the question: Are the fundamental possibilities of predicting risk at an early point in time the same for different groups of socio-economic status?

The aim of this article is twofold: Firstly, to analyse tracking of physical fitness (PF) and tracking of body mass index (BMI) in Danish adolescents from families of different socio-economic status. Secondly, to investigate whether social class differences in the prevalence of overweight and low PF exist or develop within the age range given.

## Methods

### Participants

The data were derived from the Danish part of the European Youth Heart Study (EYHS); an international study addressing cardiovascular disease risk factors in children and adolescents[15]. The study was approved by the ethic committee of Vejle and Funen.

The subjects all lived and attended school in the community of Odense in 1997 and participated in both EYHS-I, as 8–10-year-old third grade children, and in the follow-up study, EYHS-II, in 2003 as 14–16-year-old ninth grade adolescents.

### Sampling

The sampling frame was a complete list of public schools in the Municipality of Odense. Schools were stratified according to location (urban, rural) and the socio-economic character of its uptake area. From each stratum, a proportional, two-stage cluster sample of children was selected.

The primary units were the schools. Schools were selected using probability proportional to school size. Each school on the sampling list was allocated a weighting equivalent to the number of children in the school who were eligible to be selected for the study.

The secondary units were the children in the schools. Equal numbers of children were sampled from each school. Children in the appropriate age band were allocated code numbers and randomly selected using random number tables.

A more detailed description of the sampling procedure has been given elsewhere [15].

### Study sample

In all, 771 third grade children from 25 different schools were invited to participate in EYHS-I. A total number of 589 children (310 girls and 279 boys) participated in the study, corresponding to 76.4 % of the invited children.

Six years later, 384 of these children (214 girls and 170 boys) were re-examined in EYHS-II as ninth grade students, corresponding to 49.8 % of the original sample. One of the reasons for non-participation in the follow-up study was reluctance to travel to the city of Odense for the examinations by several of the participants in EYHS-I, who no longer lived in the Community of Odense at the time EYHS-II was initiated. If the coefficient of participation is calculated only taking into account subjects who still attended school in the Municipality of Odense in 2003, the coefficient rises from 49.8 % to 57.0 %.

### Representativeness

The sample of 589 participants in EYHS-I has been shown to be a representative subset of the total sample of 771 invited children with respect to physical activity level and body composition [16]. Other parameters have not been investigated.

Possible dropout effects in EYHS-II were examined by comparing baseline values between the group of children who participated in both studies and the group who only participated in EYHS-I. The socio-economic and sex-specific differences in mean BMI/PF between participants and dropouts were tested.

With respect to BMI no significant difference was found between dropouts and participants in either of the two SES groups. However, a significant dropout effect was observed in girls of high SES and in boys of low SES with respect to physical fitness. In these groups, the children who only participated in EYHS-I possessed a lower physical fitness level than the children who also participated in EYHS-II.

### Measurements

#### Physical fitness

In both studies physical fitness was determined by a progressive maximal cycle ergometer test. The protocol has been described in detail elsewhere [15]. The test was validated in both children and adolescents with a correlation coefficient of r = 0.89 and r = 0.90, respectively, to directly measured VO_2_-max. The cycle-ergometer used was a computerized Monark 839 Ergomedic, and it was programmed to increase the workload every third minute by a predefined quantity. The workload was increased until exhaustion. During the test the heart rate was monitored using a Polar Vantage NV monitor and at the time of exhaustion, the maximal heart rate and the total time were registered.

Criteria for exhaustion were:

1. A heart rate above or equal to 185 beats per minute

2. Failure to keep a pedalling frequency of at least 30 revolutions per minute

3. Subjective valuation by the test personnel

In the absence of objective cut points for low physical fitness, subjects who belonged to the lower sex and age group specific quartile of physical fitness were defined as having a low physical fitness level (Low PF).

#### Height/weight

Body height was measured to the nearest half centimetre using a stadiometer and body mass was determined by a beam-scale weight to the nearest 100 g. The subjects were only wearing underwear and a T-shirt during the examinations.

#### Body fatness

Body fatness was assessed by BMI calculations. The classification of overweight was based on BMI cut-points proposed by the International Obesity Task Force (IOTF) [17]. The cut points for overweight used included obese cut point values.

#### Pubertal stage

Assessment of biological maturation was based on Tanner's pubic hair stages for boys and Tanner's breast development stages for girls [18].

#### Socio-economic status

The classification of socio-economic status was based solely on information regarding the adult female in the household at the first measurement point, since recent evidence suggests that at least one of the risk factors considered in this study is stronger associated with the SES of the mother rather than that of the father. Two European studies on large cohorts of children have shown that the risk of being overweight in childhood/early adolescence is related to the educational level of the mother rather than that of the father [19,20]. The mothers were categorised into two different socio-economic groups. The two groups represented blue-collar and white-collar occupation respectively and they were defined by *The International Standard Classification of Occupation *scheme [21], which holds nine major categories. The categories 1–4 correspond to "blue-collar" occupation while the categories 5–9 correspond to "white-collar" occupation. Mothers working as housewives (a total number of 5 in the present sample) were classified as housekeepers (i.e. classified as belonging to the blue-collar group).

In the following text, subjects in the blue-collar group will be considered as having low SES and subjects in the white-collar group as having high SES.

### Statistics

#### Descriptive statistics

To test the difference in mean BMI/PF between groups of SES, while controlling for biological maturation state, regression analyses were applied. The corresponding differences in proportions of overweight/low PF level were tested by Fisher's exact test.

#### Tracking

The term "tracking" can be defined as: 1) the overall stability of a given variable through time (from T_1 _to T_n_). 2) the predictability of information regarding risk status at T_1 _on risk status at T_n _[22].

To assess the stability of PF and BMI throughout the measurement period, the following statistical model was used:

Yi2=β0+β1Yi1+∑j=1jβ2jXij+εi
 MathType@MTEF@5@5@+=feaafiart1ev1aaatCvAUfKttLearuWrP9MDH5MBPbIqV92AaeXatLxBI9gBaebbnrfifHhDYfgasaacH8akY=wiFfYdH8Gipec8Eeeu0xXdbba9frFj0=OqFfea0dXdd9vqai=hGuQ8kuc9pgc9s8qqaq=dirpe0xb9q8qiLsFr0=vr0=vr0dc8meaabaqaciaacaGaaeqabaqabeGadaaakeaacqWGzbqwdaWgaaWcbaGaemyAaKMaeGOmaidabeaakiabg2da9GGaciab=j7aInaaBaaaleaaieaacqGFWaamaeqaaOGaey4kaSIae8NSdi2aaSbaaSqaaiab+fdaXaqabaGccqWGzbqwdaWgaaWcbaGaemyAaKMaeGymaedabeaakiabgUcaRmaaqahabaGae8NSdi2aaSbaaSqaaGGaaiab9jdaYGqaciab8PgaQbqabaaabaGaemOAaOMaeyypa0JaeGymaedabaGaemOAaOganiabggHiLdGccqWGybawdaWgaaWcbaGaemyAaKMaemOAaOgabeaakiabgUcaRiab=v7aLnaaBaaaleaacqaFPbqAaeqaaaaa@4FA9@

where Y_i1 _is the initial observation for subject i, Y_i2 _is the second observation for subject i, β_1 _is the regression coefficient used as the stability coefficient, j is the number of time-independent covariates, X_ij _is the time-independent covariate j of individual i and ε_i _is the error term for subject i. The model can be fitted by traditional regression techniques with robust standard errors and is derived from a more general formula for assessing tracking, outlined by Twisk [23].

In order to account for scale differences, all continuous variables were transformed into subgroup specific z-scores before entering the model.

Differences between stability coefficients were tested by the following test statistics: Z=(β1∗−β2∗)(SE[β1∗])2+(SE[β2∗])2
 MathType@MTEF@5@5@+=feaafiart1ev1aaatCvAUfKttLearuWrP9MDH5MBPbIqV92AaeXatLxBI9gBaebbnrfifHhDYfgasaacH8akY=wiFfYdH8Gipec8Eeeu0xXdbba9frFj0=OqFfea0dXdd9vqai=hGuQ8kuc9pgc9s8qqaq=dirpe0xb9q8qiLsFr0=vr0=vr0dc8meaabaqaciaacaGaaeqabaqabeGadaaakeaatCvAUfeBSjuyZL2yd9gzLbvyNv2CaeHbwvMCKfMBHbaceiGaa8Nwaiabg2da9iabcIcaOGGaciab+j7aInaaDaaaleaaieaacqqFXaqmaeaacqGFxiIkaaGccqGHsislcqGFYoGydaqhaaWcbaGae0NmaidabaGae43fIOcaaOGaeiykaKYaaOaaaeaacqGGOaakcqWGtbWucqWGfbqrcqGGBbWwcqGFYoGydaqhaaWcbaGae0xmaedabaGae43fIOcaaOGaeiyxa0LaeiykaKYaaWbaaSqabeaacqaIYaGmaaGccqGHRaWkcqGGOaakcqWGtbWucqWGfbqrcqGGBbWwcqGFYoGydaqhaaWcbaGae0NmaidabaGae43fIOcaaOGaeiyxa0LaeiykaKYaaWbaaSqabeaacqaIYaGmaaaabeaaaaa@5A6C@

where β1∗
 MathType@MTEF@5@5@+=feaafiart1ev1aaatCvAUfKttLearuWrP9MDH5MBPbIqV92AaeXatLxBI9gBaebbnrfifHhDYfgasaacH8akY=wiFfYdH8Gipec8Eeeu0xXdbba9frFj0=OqFfea0dXdd9vqai=hGuQ8kuc9pgc9s8qqaq=dirpe0xb9q8qiLsFr0=vr0=vr0dc8meaabaqaciaacaGaaeqabaqabeGadaaakeaaiiGacqWFYoGydaqhaaWcbaaccaGae4xmaedabaGae83fIOcaaaaa@3059@ and β2∗
 MathType@MTEF@5@5@+=feaafiart1ev1aaatCvAUfeBSjuyZL2yd9gzLbvyNv2CaerbwvMCKfMBHbqedmvETj2BSbqee0evGueE0jxyaibaieYdOi=BH8vipeYdI8qiW7rqqrFfpeea0xe9Lq=Jc9vqaqpepm0xbbG8FasPYRqj0=yi0lXdbba9pGe9qqFf0dXdHuk9fr=xfr=xfrpiWZqaaeaabiGaaiaacaqabeaabeqacmaaaOqaaGGaciab=j7aInaaDaaaleaatCvAUfKttLearyWrP9MDH5MBPbIqV92AaGabaiab+jdaYaqaaiab=DHiQaaaaaa@3ADC@ are subgroup specific standardized regression coefficients.

To assess the predictive value of being overweight/having low PF at T_1 _on the "risk status" at T_2_, logistic regression was used. The odds ratio comparing odds of maintaining overweight/low PF during the measurement period with the odds of developing overweight/low PF at some point later than T_1_, expresses the predictability in question. Differences in predictability between groups of SES were tested by interaction terms in the logistic regression model.

Logistic regression models were also used to assess the risk of both developing and maintaining overweight/low physical fitness within the measurement period in different groups of SES.

All tracking analyses and logistic regression models were controlled for biological maturation state, and interaction terms between gender and the independent variables were added in all models to investigate whether different models should be fitted for the two sexes separately.

#### Dropout

In order to compensate for the dropout effects observed, individual measurements of PF were weighted in all tracking and logistic regression models by the inverse probability of a subject turning up at the follow-up examination given the gender, SES and the PF level at baseline [24].

## Results

Table 1 presents mean values of BMI and PF together with prevalences of overweight and low PF in 8–10-year-old children within groups of SES. Due to the significant dropout effect observed in EYHS-II, data are presented for the total sample of participants in EYHS-I. Of the 589 children who participated in EYHS-I, 42 families did not provide the necessary information for the classification of SES and 50 subjects failed to meet the criteria for exhaustion in the PF-test. In general, results showed a consistent pattern of more favourable values of the parameters in question in the group of high SES compared to the group of low SES. However, only differences in mean values of PF and in the prevalence of low PF (boys only) were significant. When interpreting the results, it is important to remember that importance should not be attached to the absolute prevalence of low PF, since the definition of low PF is based on relative cut-points. Instead, prevalences of low PF should be used only for relative comparisons between groups of SES.

Tables 2, 3, 4 present results from the longitudinal analyses performed on the 384 subjects who participated in both EYHS-I and EYHS-II. Stability coefficients of physical fitness and BMI are shown in Table 2. When comparing stability coefficients between groups of SES no statistical difference is seen. However the result indicates that the stability of PF and BMI is moderate and moderately high, respectively, independently of socio-economic position. It has been suggested that values of 0.30 to 0.60 show moderate stability while values between 0.60 to 0.90 indicate moderately high stability [25].

In Table 3 predictive values of childhood measurements of PF and BMI are presented. An odds ratio of 4.29, as seen in the group of low SES with respect to physical fitness, indicates that the odds of maintaining low PF throughout the whole measurement period is 4.29 times bigger than the odds of developing low PF at some point later than T_1_. The closer odds ratios get to 1 the lower the predictability, because the odds of maintaining risk then will be close to the odds of developing risk and, in such cases, information about risk status at T_1 _would offer no information regarding the risk profile at T_2_.

Table 4 presents odds ratios for being overweight/having low PF at the age of 14–16 years in the group of low SES compared to the group of high SES. As can be seen from the table, both the risk of being overweight and the risk of having low PF at the age of 14–16 are approximately twice as high in the group of low SES compared to the group of high SES. In order to learn about how these gradients have evolved, Table 4 also presents odds ratios for maintaining and developing risk within the measurement period. The social gradients observed at T_2 _are the combined result of the absolute prevalence of risk at T_1_, shown in Table 1, and the development and persistence of risk within the time range from T_1 _to T_2_. Both the odds of maintaining and developing overweight were more than twice as big in the group of low SES compared to the group of high SES whereas the corresponding odds for low PF were 1.36 and 1.94 times bigger in the low SES group, respectively. It should be noted, however, that only odds ratios regarding the development of overweight and low physical fitness came out significant and borderline significant, respectively.

To give an impression of the absolute probabilities from which the relative odds ratios in Tables 3 and 4 are based, Table 5 shows the raw percentages of subjects who remain "at risk" throughout the measurement period and the percentage of subjects who develop into "risk". As can be seen from the table the socioeconomic differences in the absolute probabilities are considerable of size and thus important from a public health perspective.

Interaction terms between gender and the independent variables were added in all tracking and logistic regression models and came out "not significant". Therefore, analyses were performed for the two genders simultaneously, but controlled for maturity state.

## Discussion

The first aim of the present study was to analyse tracking of physical fitness and tracking of body mass index from childhood to adolescence within low and high SES groups. In general, results showed no significant differences in tracking of PF and BMI between the two groups of SES – both the stability coefficients and the predictive values of measurements recorded in childhood did not differ significantly between the two groups. However, the stability and predictability coefficients were highly significant when calculated independent of SES on the total sample and they were moderate and moderately high for PF and BMI, respectively.

The interpretation of the size of the stability coefficients was based on predetermined cut-points distinguishing between low, moderate and high tracking effects. However, it is important to realise that the results of a tracking analysis depends on the length of the measurement period, the age of the subjects being examined, the statistical methods used, and the testing methods [26]. Thus, the cut-points used cannot be expected to hold true for all other tracking studies, and it can be difficult to compare results of tracking analyses between studies, as also pointed out by Twisk et al. [22]. No earlier study has analysed and compared tracking of CVD risk factors between children from families of different SES, thus it is not possible to compare the current findings to the work of others. Even when looking at the results independently of SES, few studies are directly comparable to our study in all of the above mentioned aspects, but despite differences in methods and ages of the populations being studied, a review of relevant literature revealed that our conclusion regarding tracking of BMI and PF, independent of SES, is in line with previous studies [11,12,27-29].

The implication of the results of the tracking analysis is that SES does not affect tracking and thereby the potential of early identification of subjects who will be unfit or overweight at a later stage in life. Furthermore, intervention programs targeting subjects who are classified as being overweight in childhood could play an essential role in the strategy for lowering the prevalence of overweight in late adolescence. In contrast, childhood measurements of PF are only moderate predictive of PF values in adolescence, resulting in more difficult conditions for identifying adolescents of low fitness at an early point in time.

The second aim of the study was to investigate whether social class differences in the prevalence of overweight and low PF exist or develop within the age range given. Longitudinal analyses showed that the onset of the development towards a social gradient in the prevalence of overweight occurs as early as during the age span of 8–16 years. As regard PF, socioeconomic differences were already present at the age of 8–10 years, indicating that the onset of the development towards a social gradient in the prevalence of low PF occurs earlier than at the age of 8–10 years.

As described in the introduction, earlier studies on the association between SES and coronary heart disease risk factors in children and adolescents have produced inconsistent results [9,10]. It has been suggested that the inconsistencies, at least partly, could be due to differences in the ages of the populations being studied [9,10]. If socioeconomic gradients do not develop until a certain stage in life is reached, naturally the association between SES and CVD risk factors will vary according to age.

Different theories have been put forward as to which stage in life the relatively clear socio-economic gradient in obesity, as well as other CVD risk factors observed in adults, start developing. Van Lenthe et al. reported no differences in biological CVD risk factors (including body fatness and PF) in both 12 and 15-year-olds but, in contrast, they observed that the low SES group had or developed a less healthy life style between 12 and 15 years of age compared to subjects in the high SES group. They hypothesised that SES differences in behavioural risk factors develop during adolescence, and will cause differences in biological risk factors to develop at the end of puberty or during early adulthood [10].

The discrepancy between the study of Van Lenthe et al. and the results of this study cannot be explained by age differences, as indirectly suggested in the study by Sobal and Stunkard [9], since socioeconomic gradients in CVD risk factors were observed in the younger of the two populations. A review of relevant literature revealed additional examples of inconsistencies between studies when taking into account the age of the subjects. For instance our cross-sectional finding that the prevalence of overweight did not differ significantly between groups of SES at the age of 8–10 years contradicts the results of another large study from a developed European country, which reported an inverse social gradient in the prevalence of overweight as early as at the age of 5–7 years [30].

A possible, but probably incomplete, explanation of the inconsistencies could be that methodological differences between studies affect the ability to detect differences in risk factors between groups of SES. For instance, different definitions of SES might not capture the factors responsible for the development of social gradients to the same extent. We tested this theory indirectly by applying a new definition of SES to our data. SES was redefined and now based on the male or female parent of the household with the highest status according to *The International Standard Classification of Occupation *scheme. In general the results showed the same overall pattern. The significant differences in low PF and insignificant differences in the prevalence of overweight, seen in Table 1, were reproduced. Also, the significant socioeconomic gradient in PF at the second measurement was maintained. However, the odds ratios of developing and being overweight at T_2 _across socioeconomic strata were no longer significant, although only small reductions were observed in the size of the odds ratios. This change in the results could very well reflect a stronger association of childhood overweight to the SES of the mother rather than that of the father, in accordance with earlier findings in the literature [19,20]. However the point is that the new definition of SES would in fact give rise to some changes in the conclusions, if the interpretations were based entirely on the observed p-values.

A further source of inconsistency between studies, when exploring at which age social gradients develop, could be differences in sample power size between studies. At which point differences between groups of SES become significant is highly dependent on sample size. For instance, the insignificant socio-economic differences in the prevalence of overweight, seen in Table 1, could in fact represent a small but true difference and thus have turned out significant given a higher power.

Finally, it can be questioned whether differences between groups of SES emerge at the exact same age in all developed countries or if individual differences exist between developed countries, analogous to the fact that the association between SES and CVD risk factors differs between developed and developing countries [31,32].

To which extent these methodological issues can account for previous inconsistent findings regarding the association between SES and CVD risk factors, is without doubt an important research question for future studies. Consistent information about the timing and development of socio-economic gradients in CVD risk factors is important to help set the scene for more detailed research into the possible physiological and psychosocial mechanisms responsible for the socio-economic inequalities.

## Conclusion

In conclusion, tracking of PF and BMI from childhood to mid-adolescence is not significantly different between groups of SES, thus indicating that the fundamental possibilities of predicting risk at an early point in time are the same for different socio-economic status groups.

However, social inequalities in the absolute prevalence of overweight and low PF are developing or already present during this particular age period. Results revealed the presence of a social gradient in the prevalence of low PF at the age of 8–10 years, favouring the children of high socioeconomic status and demonstrated that a social gradient in the prevalence of overweight develops no later than during the age period from 8–16 years, partly due to a higher incidence of overweight in the low SES group. These results underline the need for preventive efforts targeting children of low SES in early childhood and furthermore indicate that efforts should not be based solely on the principle of tracking (i.e. aimed exclusively at children who are already classified as being "at risk" at the time given), but initiatives should also be taken to lower the occurrence of new cases of overweight and low PF in early childhood within the group of low SES.

## Competing interests

The author(s) declare that they have no competing interests.

## Authors' contributions

NCM has contributed to the analysis and interpretation of data and in preparing the manuscript. CNB has contributed to the acquisition of data and in preparing the manuscript. KF, NW and LBA participated in the design of the study and contributed to the analysis and interpretation of data. PLK performed the statistical analysis and interpretation of data, contributed to the acquisition of data and wrote the paper. PLK revised it with contributions from all authors. All authors read and approved the final manuscript.

## Pre-publication history

The pre-publication history for this paper can be accessed here:



## Figures and Tables

**Table 1:** Mean and prevalence of CVD risk factors by socio-economic status in 8–10-year-old children

	**Low SES**			**High SES**		
		
	**Total group size**	**At-risk prevalence % (95 % CI)**	**Mean (SD)**	**Total group size**	**At-risk prevalence % (95 % CI)**	**Mean (SD)**
**Physical fitness**						
**Girls**	107	30.84^† ^(22.27–40.50)	8.75^‡ ^(1.41)	160	21.25^† ^(15.19–28.41)	9.15^‡ ^(1.39)
**Boys**	114	32.46^‡ ^(23.99–41.86)	9.94^‡ ^(1.74)	122	15.57^‡ ^(9.64–23.25)	10.50^‡ ^(1.48)
						
**Body Mass Index**						
**Girls**	124	16.13 (10.34–23.81)	17.43 (2.63)	168	13.69 (8.88–19.83)	17.08 (2.45)
**Boys**	124	15.32 (9.48–22.89)	17.48 (2.71)	131	12.98 (7.74–19.96)	17.28 (2.23)

Subjects were classified as being at-risk in relation to physical fitness, if they belonged to the lower sex and age group specific quartile of physical fitness. In relation to BMI, subjects were classified as being at-risk if they were overweight according to BMI cut-points proposed by the International Obesity Task Force. Physical fitness was measured in watt/kg^2/3 ^and BMI in kg/m^2^

‡ Significantly different from the corresponding value in the opposite SES group (P < 0.05)

† Borderline significantly different from the corresponding value in the opposite SES group (0.05< P < 0.09)

**Table 2:** Stability (tracking) coefficients β adjusted for maturity state and gender, reported for different groups of SES separately and for the total sample

	**Low SES**			**High SES**			**Total sample**			
	**n**	**β**	**95% CI**	**n**	**β**	**95% CI**	**n**	**β**	**95% CI**	**P**^a^
**Physical fitness**	133	0.45	0.25–0.64	195	0.54	0.41–0.67	328	0.50	0.38–0.61	0.54
**Body Mass Index**	161	0.75	0.61–0.90	219	0.70	0.60–0.80	380	0.73	0.64–0.81	0.60

a: P-value for the test of whether stability coefficients differs between the two groups of SES

**Table 3:** Predictability odds ratios adjusted for maturity state and gender, reported for the two groups of SES separately and for the total sample

	**Low SES**			**High SES**			**Total sample**			
	**n**	**OR**	**95 % CI**	**n**	**OR**	**95 % CI**	**n**	**OR**	**95 % CI**	**P**^a^
**Physical fitness**	133	4.29	1.85–9.93	195	7.31	3.20–16.71	328	5.44	3.03–9.75	0.47
**Body Mass Index**	161	15.73	5.26–47.03	219	17.08	5.41–53.92	380	16.26	7.40–35.80	0.89

a: P-value for the test of whether odds ratios differs between the two groups of SES

**Table 4:** Odds ratios for specific risk events in the group of low SES compared to the group of high SES. Odds ratios are adjusted for maturity state and gender

	**n**	**OR**	**95% CI**	**P**^a^
**Physical Fitness**				
Being low fit at T_2_	328	1.77	1.04–3.01	0.034
Maintaining low PF	81	1.36	0.55–3.36	0.511
Developing low PF	247	1.94	0.95–3.95	0.069
				
**Body Mass Index**				
Being overweight at T_2_	380	1.95	1.07–3.57	0.030
Maintaining overweight	46	2.09	0.60–7.26	0.245
Developing overweight	334	2.35	1.02–5.40	0.045

Low physical fitness was defined according to the lowest sex and age group specific quartile of physical fitness. Overweight was defined according to BMI cut-points proposed by the International Obesity Task Force.

a: P-value for the test of whether odds ratios differs from 1

**Table 5:** Unadjusted frequencies of subjects maintaining or developing risk within the measurement period. The probabilities are weighted for dropout.

	**Low SES**		**High SES**		**Total sample**	
	**Maintain (n)**	**Develop (n)**	**Maintain (n)**	**Develop (n)**	**Maintain (n)**	**Develop (n)**
			
**Low PF**	55.48 % (38)	21.36 % (95)	47.76 % (43)	12.28 % (152)	51.82% (81)	15.89 % (247)
**Overweight**	69.59 % (22)	12.52 % (139)	51.72 % (24)	5.41 % (195)	60.31 % (46)	8.60 % (334)

Low physical fitness was defined according to the lowest sex and age group specific quartile of physical fitness. Overweight was defined according to BMI cut-points proposed by the International Obesity Task Force.

n denotes the number of subjects on which the percentages are based
